# Reconfiguring local characteristics in contemporary Muju: an AI-assisted study of vocal style in Chinese local opera

**DOI:** 10.3389/fpsyg.2026.1856609

**Published:** 2026-07-02

**Authors:** Yijia Cao, Hong Liu, Tianlun Luo

**Affiliations:** 1 Cai Yuanpei College of Art and Design, Shaoxing University, Shaoxing, China; 2 Department of Musicology, Shanghai Conservatory of Music, Shanghai, China; 3 School of Advanced Technology, Xi'an Jiaotong-Liverpool University, Suzhou, China

**Keywords:** AI-assisted analysis, Chinese traditional opera, Muju, cross-genre influence, training transfer

## Abstract

**Background:**

Muju (睦剧) is a local opera rooted in Chun’an County, Zhejiang Province, China. Since the re-establishment of its professional troupe in 2015, its vocal style has changed substantially, driven by the recruitment of young performers trained in other opera genres (Huangmeixi, Yueju, Wuju). Previous research has described this change through historical narratives and insider accounts, but the extent to which cross-genre training may shape vocal production has remained insufficiently examined with empirical methods.

**Objective:**

This study examines how performers’ prior vocal training in other operatic genres may affect vocal production when performing Muju, and how this process may have contributed to changes in the genre’s local vocal characteristics over time.

**Methods:**

A total of 691 vocal excerpts were analyzed, including 196 Muju excerpts across three historical stages and 495 reference excerpts from Yueju, Huangmeixi, and Wuju. A three-layer Transformer-decoder model was used as part of an AI-assisted, score-based acoustic-proximity analysis. The model-derived scores were treated as exploratory indices of relative acoustic proximity rather than as validated measures of stylistic similarity. In addition, a case-based acoustic comparison of the same aria was conducted among three performances: a historical male Muju/Sanjiaoxi reference, a contemporary Huangmeixi-trained female performer, and a contemporary Chun’an local female reference.

**Results:**

Model-derived scores suggest that Contemporary Muju may show lower relative proximity to Traditional Sanjiaoxi than Old Muju does (Cohen’s *d* = −0.66), along with stronger Huangmeixi-related score tendencies across historical stages. Performer-level results further suggest variation in Yueju- and Wuju-related model-derived scores by training background. At the case level, the acoustic comparison suggests recurrent differences in vowel openness and articulatory placement among the three performances, especially in the contemporary same-gender contrast between P1 and P7.

**Conclusion:**

Taken together, the findings are consistent with the possibility that the vocal profile of contemporary Muju has been reconfigured rather than replaced. They are also compatible with the possibility that cross-genre training contributed to this process through the transfer of habitual vocal-production patterns. The acoustic case study provides a focused performance-level illustration, whereas the AI-assisted score-based analysis offers exploratory corpus-level context requiring cautious interpretation.

## 1 Introduction

### 1.1 Vocal style change in local opera: the case of Muju

When performers trained in one vocal tradition begin performing in another, do their previously acquired vocal habits persist and reshape the sound of the target genre? This question has broad relevance for the study of musical performance, yet it has rarely been investigated with quantitative methods in the context of traditional opera. The ongoing transformation of Muju (睦剧), a local opera genre rooted in Chun’an County, Zhejiang Province, China, offers a particularly revealing lens through which to examine this very dynamic.

The origins of Muju trace back to Sanjiaoxi (三脚戏), a folk song-and-dance form typically performed by three role types: sheng (生), dan (旦), and chou (丑). In the early 1950s, with the establishment of a professional troupe, the name “Muju” was formally adopted, and the genre entered a new stage of institutionalized development. Existing studies suggest that Muju developed through sustained interaction with Huangmeixi (黄梅戏) and other adjacent operatic traditions (Hong, 1982; Luo, 2007; Jiang and Zhou, 1998; Chen, 2009; Gao and Jin, 2015). Muju is thus locally rooted yet historically open in its stylistic formation.

The contemporary transformation of Muju has become especially visible since the re-establishment of the Chun’an County Muju Troupe in 2015. After the dissolution of the professional ensemble in the late 1980s, Chun’an had no professional Muju troupe for more than two decades. When the troupe was reorganized in 2015, many former performers were no longer able to serve as principal stage actors due to age, and economic factors deterred local young people from entering the profession. Under these circumstances, the troupe recruited performers from opera vocational schools in Zhejiang and neighboring provinces. Many of these performers had been trained in Huangmeixi, Yueju (越剧), or Wuju (婺剧). These performers brought with them vocal habits formed through years of institutionalized training in other genres. As a result, the same Muju opera may sound stylistically different depending on who is singing. What is changing is not only the personnel structure of the troupe, but also the vocal style of Muju itself.

### 1.2 Cross-genre training and the need for empirical evidence

Previous studies have described Muju’s vocal-style change at the level of insider description, especially those of local audiences and senior performers. Such accounts preserve local criteria for recognizing what counts as “Muju,” but they do not by themselves clarify the degree, manner, or mechanism of stylistic change (Gao and Jin, 2015; Chen, 2009).

What remains unclear is whether the current state of stylistic change in Muju reflects long-term historical affinity, more recent reconfiguration under contemporary performance conditions, or a combination of both. A more explicit analytical framework is needed to assess the extent to which Contemporary Muju departs from Traditional Sanjiaoxi, aligns with neighboring genres, and registers traces of cross-genre training in measurable acoustic terms.

In performance research, this gap is particularly significant. Discussions of change in performing arts traditions often rely on repertoire analysis, institutional history, or performer interviews. By contrast, the acoustic dimensions of vocal production—how sound is produced and how production patterns differ across training backgrounds—remain underexamined. This matters because vocal style in operatic singing is not only a matter of repertoire or notation, but also of embodied vocal-production habits that performers carry from their training into performance.

Existing research leaves a gap at the intersection of three bodies of literature. Local historical and insider studies of Muju have clarified the genre’s origins, repertoire, institutional development, and local understandings of stylistic change. However, they rarely examine how such change is realized in vocal production. Performance-science studies of motor learning and vocal expertise explain how training can produce stable embodied habits, but they have seldom addressed the reconstitution of Chinese local opera traditions under contemporary performer mobility. Meanwhile, AI-assisted music-classification studies have demonstrated the feasibility of modeling genre-related acoustic patterns. Yet they have generally focused on intergeneric classification rather than on how within-genre vocal variation relates to performers’ training backgrounds. The present study is positioned at the intersection of these approaches. It uses performance-science theory to frame cross-genre training as embodied vocal transfer. It then uses acoustic and computational methods to examine how such transfer may become audible in Muju vocal production.

### 1.3 Training transfer in embodied vocal production: a performance science framework

The phenomenon observed in contemporary Muju, where performers trained in one system produce sound in another, can be understood through the lens of training transfer, a concept central to the study of motor learning and expertise. Research on motor skill acquisition has shown that practiced movement patterns often persist when performers enter new task environments. This phenomenon has been described as transfer of training, motor habit persistence, or interference effects (Schmidt et al., 2019; Magill and Anderson, 2016). In the performing arts, analogous processes have been documented in dance. Neuroimaging research has shown that motor representations are style-specific: dancers show stronger premotor activation when observing movements from their own trained style than from an unfamiliar one (Calvo-Merino et al., 2005). Moreover, movement patterns acquired through prolonged training in one dance style can produce proactive interference when dancers attempt different movement approaches (Karin, 2016).

In vocal performance, the relevance of training transfer is well supported by research on singing expertise. Vocal training produces lasting changes in habitual patterns of articulatory placement, vocal-tract shaping, and resonance strategy (Sundberg, 1987; Titze, 2000). Studies comparing trained and untrained singers have demonstrated measurable differences in spectral energy distribution, particularly in the 2–4 kHz singer’s formant region, that reflect long-term vocal adaptation rather than momentary choices (Sundberg, 1987; Omori et al., 1996). Longitudinal evidence further indicates that vocal training progressively reshapes acoustic parameters such as fundamental frequency range and intensity over successive semesters of study (Mendes et al., 2003). More recent work on Peking opera has likewise shown that vocal timbre functions as an analytically meaningful dimension of role and style differentiation, further indicating that vocal production in operatic traditions carries genre-specific acoustic signatures shaped by training (Liu and Wallmark, 2024).

These findings suggest that when a performer trained in one opera genre begins to sing in another, their vocal output may retain acoustic traces of prior training, manifested in habitual patterns of vowel shaping, articulatory placement, and resonance-related tendencies. The Muju case provides a particularly clear context for examining this proposition. Its recent reconstitution involved the systematic incorporation of performers with known and documented training backgrounds in neighboring genres. This makes it possible to examine training-related vocal differences within a naturalistic performance setting.

At the same time, the feasibility of using computational methods to study such cross-genre vocal differences is supported by a growing body of research on AI-assisted music classification, including recent work specifically on Chinese opera genres (Zhang et al., 2021; Li and Hu, 2023). However, while previous studies have explored intergeneric classification, they have not examined how within-genre vocal variation relates to individual performers’ training backgrounds—a gap the present study seeks to address.

Taken together, the present study adopts training transfer in embodied vocal production as its guiding theoretical framework. In this framework, genre-specific vocal training is understood not merely as the acquisition of repertoire knowledge, but as the formation of relatively stable vocal-production habits, including vocal-tract configuration, articulatory placement, resonance strategy, and timbral shaping. When performers trained in one opera genre enter another genre, these embodied vocal habits may persist, adapt, or interfere with the target tradition, thereby producing measurable acoustic tendencies without necessarily replacing the target genre. This framework guides the present research design. The corpus-level AI-assisted analysis explores patterned acoustic tendencies across genres and historical stages. The case-based acoustic analysis then examines how such tendencies may become audible in concrete vocal production.

### 1.4 Research aims and analytical approach

Against this background, the present study investigates how the local vocal characteristics of Muju may have been reconfigured in contemporary performance under the influence of cross-genre-trained performers. More specifically, it addresses three questions:

*RQ1*: Compared with Traditional Sanjiaoxi and Old Muju, does Contemporary Muju show higher model-derived relative acoustic-proximity scores in relation to neighboring genres such as Huangmeixi, Yueju, and Wuju?

*RQ2*: Does Contemporary Muju show lower model-derived relative acoustic-proximity scores in relation to Traditional Sanjiaoxi than Old Muju does?

*RQ3*: Can possible traces of cross-genre training be observed in the acoustic details of vocal production by performers with different training backgrounds?

These questions translate the theoretical framework of training transfer into two empirical levels: RQ1 and RQ2 examine corpus-level patterns of acoustic alignment and divergence, whereas RQ3 examines whether possible training-related transfer can be observed in the acoustic details of vocal production.

To address these questions, the study adopts a mixed-method approach. First, a three-layer Transformer-decoder model is used as part of an AI-assisted, score-based acoustic-proximity analysis to generate model-derived scores across historical periods and genres. Second, a case-based acoustic comparison of the same Muju aria examines whether possible training-related differences can be observed in concrete vocal production. The former provides exploratory corpus-level context, whereas the latter offers a focused performance-level illustration.

## 2 Materials and methods

### 2.1 Corpus construction and data sources

The dataset comprised one Muju corpus and three reference corpora drawn from neighboring opera genres. The Muju corpus contained 196 vocal excerpts, divided into three historical stages: Traditional Sanjiaoxi, Old Muju, and Contemporary Muju. The reference corpora consisted of 166 Yueju excerpts, 162 Huangmeixi excerpts, and 167 Wuju excerpts. These three genres were selected because each either shares historical affinities with Muju or has influenced contemporary Muju through the recruitment of performers and musicians. All three also developed in geographical proximity to Chun’an County and have had recognizable cultural influence within the county.

The Muju corpus was divided into three historical stages on the basis of institutional organization and performance form. The Traditional Sanjiaoxi period refers to materials predating 1951, representing the early tradition of Muju vocal practice. The Old Muju period denotes the institutionalized phase from the 1950s to the 1980s—a designation employed by insiders within the tradition. The Contemporary Muju period encompasses materials produced after the professional troupe was formally reestablished in 2015 (Table 1).

**Table 1:** Corpus composition and data sources.

Corpus	Number of excerpts	Main source type(s)
Muju (total)	196	Archival and field recordings
Yueju	166	Published recordings
Huangmeixi	162	Published recordings
Wuju	167	Published recordings
Grand total	691	—

The Muju corpus was drawn from archival materials and field recordings collected by the first author in Chun’an County between 2019 and 2025. The Yueju, Huangmeixi, and Wuju corpora were drawn from publicly available publications. The recordings were selected according to four criteria: recording quality, temporal coverage, representativeness of the excerpts, and structural balance across role types, tune types, and repertoire genres. For the three reference corpora, the selected recordings span from the 1940s to the 2020s. The excerpts consist predominantly of canonical arias drawn from representative repertoire of each genre, performed by widely recognized artists from different periods (e.g., Wenjuan Wang, Ruijuan Fan, and Weitao Mao for Yueju). To ensure coverage of systematic stylistic variation, the corpora were balanced across major vocal-tune categories, role types, and rhythmic types. For instance, the Wuju corpus includes excerpts from six distinct vocal-tune systems (e.g., Gaoqiang, Huidiao, Kunqiang), with comparable representation of dan and sheng roles.

All recordings were manually segmented into excerpts using audio-editing software (REAPER). Each excerpt was assigned a unique numerical identifier and accompanied by metadata including opera title, aria title, role type, lyrics, and, where applicable, tune name, tonal designation, and rhythmic type.

Performer identity was documented as part of corpus management. This was particularly important because the present study examines the relationship between cross-genre training and vocal style. According to the first author’s field investigation, when the Chun’an County Muju Troupe was officially re-established on December 31, 2015, it had a total of 19 actors, among whom only two of the younger performers were natives of Chun’an.

Within the Contemporary Muju corpus, five performers were identified as either non-local or possessing substantial prior training in other opera genres. Among the 85 excerpts, 38 were sung by local Chun’an Muju performers, 21 by Huangmeixi-trained performers (2 singers), 18 by Yueju-trained performers (2 singers), and 8 by a Wuju-trained performer (1 singer). Performer identity was not entered into the model as a classification label; instead, it was retained in the corpus documentation to support later interpretation of the relationship between training background and vocal style (Table 2).

**Table 2:** Performer backgrounds and excerpt counts in the contemporary Muju corpus.

Performer ID	Hometown	Prior genre training	Formal institutional training (Y/N)	Role type	Number of excerpts
P1	Anqing, Anhui	Huangmeixi	Y	dan	18
P2	Chun’an, Zhejiang	Huangmeixi	Y	dan	7
P3	Yuyao, Zhejiang	Yueju	Y	sheng	12
P4	Ningbo, Zhejiang	Yueju	N	sheng	6
P5	Jinhua, Zhejiang	Wuju	Y	sheng	8

Co-performed excerpts were counted for both performers.

It should be noted that the Traditional Sanjiaoxi corpus is limited to 20 excerpts, substantially smaller than the Old Muju (91) and Contemporary Muju (85) corpora. This imbalance reflects the scarcity of surviving historical audio materials rather than a selective sampling decision. Muju/Sanjiaoxi is a rare and under-documented local opera tradition, and very few early recordings have been preserved. The first author therefore searched and organized the accessible audio materials representing the pre-1951 Sanjiaoxi tradition through local archives and senior practitioners. To the best of our knowledge, no previous study of Sanjiaoxi/Muju has incorporated surviving audio recordings as primary research materials. Existing studies have relied mainly on historical description, local documentation, repertoire accounts, or insider narratives.

Because the Traditional Sanjiaoxi corpus functions as a key historical reference in several comparisons, results involving this corpus may be more sensitive to individual recordings than comparisons based on larger corpora. For this reason, comparisons involving Traditional Sanjiaoxi are treated as preliminary and exploratory tendencies rather than as stable estimates of historical-stage differences. The implications of this imbalance for statistical comparison are further addressed in the Statistical Analysis and Limitations sections.

### 2.2 AI-assisted score-based acoustic-proximity analysis

#### 2.2.1 Experimental design

The training-transfer framework directly shaped the comparative logic of the AI-assisted analysis. If performers’ prior genre training leaves residual traces in Muju vocal production, such traces would not necessarily appear as complete stylistic replacement, but may appear as patterned score tendencies toward the genres in which performers were previously trained, or may appear as reduced alignment with earlier local Muju/Sanjiaoxi vocal practice. Accordingly, the external comparisons were designed to examine whether Muju excerpts from different historical stages show differing model-derived score tendencies in relation to Huangmeixi, Yueju, and Wuju. The internal comparisons were designed to examine whether Contemporary Muju shows weaker model-derived alignment with Traditional Sanjiaoxi than Old Muju does. These comparisons are therefore not treated as direct tests of stylistic identity, but as exploratory operationalizations of possible training-related acoustic transfer at the corpus level.

On this basis, the analysis was organized into external and internal comparisons.

In the external comparison, Huangmeixi, Yueju, and Wuju served as reference corpora, whereas the three Muju stages served as target corpora. This design estimated how Traditional Sanjiaoxi, Old Muju, and Contemporary Muju related to neighboring genres. In the internal comparison, Traditional Sanjiaoxi served as the reference corpus, and Old Muju and Contemporary Muju served as the target corpora, allowing assessment of diachronic divergence from earlier local style.

The analysis consisted of five comparative settings grouped into two families: three external comparisons and two internal comparisons. This experimental structure allowed the study to address the same object—Muju—from two complementary angles. The model was therefore not used simply to identify “the most similar genre,” but to construct a comparative framework for examining stylistic reconfiguration across periods and across genres.

The purpose of this design was not to build a standalone automatic genre-classification system. Rather, it was to construct a score-based comparative procedure for examining vocal-production tendencies across corpora. In the present study, supervised classification was used as a modeling step through which the model learned genre-discriminative acoustic patterns from reference labels. The resulting values were then treated as model-derived scores indicating the degree to which a given excerpt was associated with particular learned acoustic patterns within the model’s feature space.

In this operational sense, “relative acoustic proximity” refers to a score-based association between an excerpt and a learned genre-related acoustic pattern. It should not be understood as a direct measure of genre identity or validated stylistic similarity. Rather, it provides an exploratory acoustic basis for discussing stylistic tendencies at the level of vocal production. This interpretation is appropriate to the present research context because Muju practitioners and local insiders often understand vocal timbre, articulation, resonance, and habitual singing features as audible components of genre style.

#### 2.2.2 Model architecture

The use of AI-assisted classification in this study builds on a well-established research tradition in music information retrieval (MIR). Since the influential work of Tzanetakis and Cook (2002), audio-based genre classification has shown that machine learning can model stylistic similarity and difference on the basis of acoustic features. More directly relevant to the present study, recent work has applied convolutional neural networks (Zhang et al., 2021) and joint time-and-frequency transformer models (Li and Hu, 2023) to Chinese opera classification, providing a preliminary methodological basis for the present approach.

The core model was based on a Transformer decoder architecture. Before model training, all audio recordings were converted to mono waveforms and then transformed into Mel-frequency cepstral coefficients (MFCCs), which served as the primary representation of the acoustic signal.

After MFCC extraction, the input features were passed through a down-sampling module for dimensionality reduction and preliminary feature refinement. This module consisted of three one-dimensional convolutional layers with kernel sizes of 1 × 15, 1 × 7, and 1 × 1, respectively. Each convolutional layer was followed by max pooling and batch normalization. This design was adopted to improve feature extraction under conditions of limited training data while preserving sufficient temporal and spectral information for the subsequent score-based acoustic-proximity analysis.

The Transformer decoder was adapted from the model proposed by Vaswani et al. (2017), but the original six-layer configuration was reduced to three layers to better match the size of the present corpus. To reduce interference from heterogeneous musicological taxonomies across genres, the experiments adopted a minimal labeling strategy: only opera genre identity was provided to the model during training and testing. This decision was methodologically important because the four genres under comparison differ considerably in their internal terminologies and classification systems. A more elaborate cross-genre label system might have imposed artificial equivalences and encouraged the model to rely on prior categorical assumptions rather than on stylistic evidence contained in the recordings themselves.

**Figure 1:**
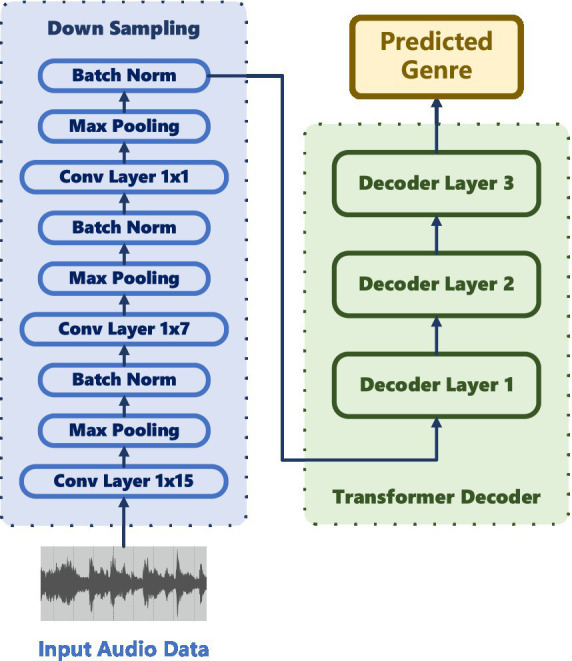
AI model architecture. The diagram shows the feature-learning pipeline used in the score-based acoustic-proximity analysis. Mono audio input was transformed into MFCC features, passed through a convolutional down-sampling module, and then processed by a three-layer Transformer decoder. The final output was used to generate genre-label scores, which were interpreted as model-derived acoustic-proximity scores rather than as direct measures of stylistic similarity.

Figure 1 summarizes the model pipeline from mono audio input and MFCC extraction to convolutional down-sampling, Transformer-decoder feature learning, and genre-label scoring. The figure is intended as a schematic representation of the feature-learning architecture, whereas the two-phase training and fine-tuning procedure is described separately in Section 2.2.3.

#### 2.2.3 Model training

The model was trained in two phases. In Phase 1 (pre-training), the 691 excerpts formed a six-category pre-training corpus, on which the model was trained from scratch as a 6-class classifier for 50 epochs. This corpus was divided into stratified training and validation subsets at an 85/15 ratio, with stratification conducted at the genre-label level. Training used the AdamW optimizer (β_1_ = 0.9, β_2_ = 0.999, weight_decay = 1 × 10^−4^), a cosine annealing learning-rate schedule from 1 × 10^−4^ to 1 × 10^−6^, and a batch size of 192. The purpose of this phase was to learn broad genre-discriminative acoustic representations for subsequent score-based comparison, rather than to provide an independent estimate of out-of-sample classification performance.

In Phase 2 (fine-tuning), starting from the epoch-50 pre-trained checkpoint, the 6-class prediction head was replaced with a randomly initialized 2-class linear head (Xavier uniform initialization). The model was then fine-tuned separately for each binary classification task with a fixed learning rate of 1 × 10^−6^ and the same optimizer configuration.

For the score-based analyses reported in Sections 3.1.1–3.1.3, the epoch-15 checkpoint was used. Fine-tuning performance was additionally monitored up to epoch 20 as an internal diagnostic check to examine whether additional training improved performance beyond the epoch-15 checkpoint.

A stratified 70/15/15 train/validation/test split was used in the fine-tuning stage. Stratification was conducted at the genre-label level rather than at the performer level. Performer identity was not used as a model input or classification label, but it was retained as metadata for later interpretation of performer-level variation. This partitioning strategy should therefore be understood as genre-label stratification, not as performer-independent validation. Because the same performer could in principle appear in more than one data split, the evaluation metrics reported in Section 3.1.4 should not be interpreted as evidence of speaker-independent classification performance.

This distinction is important for the present study. The purpose of the model was not to build a standalone optimized genre-classification system, but to construct a score-based comparative procedure for examining relative acoustic-proximity tendencies. The model-derived scores may reflect multiple factors, including acoustic regularities relevant to genre, properties of the training data, performer-specific vocal characteristics, and the structure of the learned feature space. For this reason, the statistical analyses applied to these scores are used only to assess whether group differences in model-derived scores are patterned across corpora or performers. These scores are not treated as classification labels or as validated measures of stylistic similarity. The interpretive limits of this procedure are discussed further in Section 4.4.

To provide a basic diagnostic check of whether the model learned genre-discriminative acoustic patterns under the present corpus partitioning, internal evaluation metrics were computed for the three reference genres with the largest sample sizes: Huangmeixi (*n* = 162), Wuju (*n* = 167), and Yueju (*n* = 166). The Muju corpus also contains a substantial total number of excerpts (*n* = 196). However, it comprises three historically distinct sub-categories: Traditional Sanjiaoxi (*n* = 20), Old Muju (*n* = 91), and Contemporary Muju (*n* = 85). Because each sub-category has a limited sample size, per-subcategory evaluation would be unstable. Treating Muju as a single aggregated evaluation class would also be methodologically inappropriate. The diagnostic results, including training loss curves, validation accuracy, and test accuracy under the present partitioning, are reported in Section 3.1.4.

### 2.3 Acoustic case analysis

Whereas the AI-assisted analysis operationalizes training transfer at the corpus level through model-derived score tendencies, the acoustic case analysis examines the same theoretical issue at the level of concrete vocal production. Under the training-transfer framework, possible transfer is expected to appear not as a wholesale change of repertoire, but as recurrent differences in vowel shaping, articulatory placement, and related acoustic features when performers with different training backgrounds sing the same aria.

#### 2.3.1 Case selection

Accordingly, the study includes a case-based acoustic comparison designed to examine how cross-genre training becomes audible in specific Muju performances. The analysis focuses on the aria “Zheng yue shi xin nian” (正月是新年) from the Muju small play *Nanshan Zhongmai* (《南山种麦》). This aria was selected for two reasons. First, it is highly representative: *Nanshan Zhongmai* is a canonical Muju play performed continuously from the Sanjiaoxi period to the present, and “Zheng yue shi xin nian” remains its most widely circulated aria. Second, the aria exhibits considerable stability in both lyrical content and melodic framework, making it well suited for cross-period comparison and precise syllable-by-syllable alignment.

Three performances of this aria were selected for comparison. The first was recorded in the 1950s and sung by P6, a representative performer whose career spanned both the Sanjiaoxi period and the Muju period. P6’s father had founded a Sanjiaoxi troupe in Chun’an County, and P6 grew up immersed in the sound world of Sanjiaoxi. His recording is one of his best-known performances and also one of the earliest surviving recordings of Muju. In the present study, P6 is retained as a historical reference for traditional Muju/Sanjiaoxi vocal practice.

The second performance was recorded by the first author in Chun’an in 2022 and sung by P1. P1, a native of Anqing, Anhui Province, had undergone long-term formal training in Huangmeixi and graduated from Anhui Huangmeixi Vocational Art College. After joining the Chun’an County Muju Troupe in 2015, she became one of its leading performers. P1 is therefore selected as a contemporary Muju performer whose vocal production may reflect cross-genre training.

The third performance was also recorded by the first author in Chun’an in 2022 and sung by P7. P7 is a female Chun’an local performer who had not received professional opera-school training. She is included as a contemporary same-gender local reference for Muju vocal practice.

The inclusion of P7 is methodologically important because a comparison between P1 and P6 alone would conflate possible training-related differences with differences in gender, historical period, and recording condition. In the present design, P6 is therefore retained primarily as a historical reference for earlier Muju/Sanjiaoxi vocal practice, whereas P7 provides a contemporary same-gender local reference. The P1–P7 comparison forms the central contemporary contrast for examining whether P1’s vocal production shows patterns compatible with possible effects of prior Huangmeixi training.

All three performances present the same aria under different historical and training conditions. The contemporary singers were recorded in a relatively controlled solo format and were asked to maintain textual completeness, overall pitch level, and tempo stability so that their versions would remain as comparable as possible to the historical reference. All three versions are unaccompanied or minimally affected by instrumental texture, which reduces the influence of accompaniment on formant and spectral-envelope analysis. Nevertheless, full equivalence among the recordings is not achievable, given differences in recording period, performer background, and recording condition. The case comparison is therefore interpreted conservatively.

#### 2.3.2 Acoustic processing and analysis

Acoustic analysis was conducted in Python using the Librosa and Parselmouth libraries. All audio files were converted to mono, resampled to 22,050 Hz, and silence-trimmed before analysis. The first three formants (F1–F3) were extracted using the Burg algorithm with a time step of 0.01 s. Comparative LPC spectral envelopes were also generated using Linear Predictive Coding (order 16), and all figures were produced using Matplotlib.

Because the analysis focuses on short syllable excerpts, additional cleaning procedures were applied to reduce the influence of segment boundaries and automatic formant-tracking artifacts. For each syllable excerpt containing *N* formant frames, the central portion was operationally defined as the frame-index range from floor (0.20 *N*) to ceil (0.80 *N*) − 1. This step reduced the influence of unstable onset and offset portions, which may contain consonantal transitions, breath noise, or sliding movement. After this temporal trimming, implausible formant values were treated as missing values using singer-specific thresholds. For the two female singers, P1 and P7, conservative thresholds were set at F1 = 200–1,200 Hz, F2 = 600–3,500 Hz, and F3 = 1,200–4,500 Hz. For the male historical reference P6, thresholds were set at F1 = 150–1,100 Hz, F2 = 500–3,200 Hz, and F3 = 1,000–4,200 Hz. Missing values were omitted from calculations of means and standard deviations.

After this cleaning procedure, raw F1–F3 means and standard deviations were recalculated for the matched syllable excerpts. In addition to raw formant values, within-singer z-score normalization was applied. The normalization was performed separately for each singer and each formant dimension. It was calculated across the cleaned frames from the selected syllable excerpts. The normalized values were used as a descriptive sensitivity check to examine whether observed patterns remained visible after reducing singer-specific scaling effects. Because the normalization was based on selected syllable excerpts rather than on a full vowel inventory, it is not used for population-level inference.

The reported acoustic interpretation focuses primarily on two formant-based dimensions of vocal production: vowel openness and articulatory placement. Higher F1 values were interpreted as indicating greater vowel openness, whereas F2 patterns were used to infer relatively fronted or backed articulation, following standard principles in acoustic phonetics (Stevens, 1998; Ladefoged and Johnson, 2014).

Although F3 values and LPC spectral envelopes were generated during acoustic processing, they are used only as ancillary descriptive materials in the present analysis. Because the revised case comparison does not provide a systematic F3/LPC-based analysis across all three performers, the main interpretation does not make independent claims about resonance organization. References to vocal timbre and resonance are therefore limited to the broader theoretical understanding that vowel shaping and articulatory placement contribute to habitual vocal production in genre-specific singing (Sundberg, 1987; Titze, 2000; Titze et al., 2017).

It should be clarified that formant-related differences are not treated here as direct equivalents of “style.” Rather, they are used as acoustic indicators of recurrent differences in vocal production. In genre-specific theatrical vocal traditions, stable patterns of vowel shaping, articulatory placement, vocal-tract configuration, and resonance adjustment may contribute to timbral profile and habitual ways of sound production. In the present case analysis, however, the evidential basis rests mainly on F1 and F2 patterns across matched syllables. These patterns are used to document production-related features through which possible training-related differences may become acoustically traceable.

The acoustic analysis was designed as a focused case study rather than a large-sample experiment. Its purpose was descriptive and comparative: to examine whether P1, a Huangmeixi-trained contemporary Muju performer, exhibits vocal-production patterns that differ from both a historical Muju/Sanjiaoxi reference (P6) and a contemporary Chun’an local female reference (P7). The P1–P6 comparison is treated primarily as a historical reference because it involves differences in gender, recording period, and recording condition. The P1–P7 comparison provides the central contemporary contrast for examining whether P1’s vocal production shows patterns compatible with possible effects of prior Huangmeixi training. The acoustic case analysis therefore functions as a micro-level complement to the corpus-level AI-assisted analysis, not as definitive proof of a causal training effect.

### 2.4 Statistical analysis

Group comparisons of model-derived acoustic-proximity scores were conducted as exploratory statistical summaries rather than as confirmatory hypothesis tests. Welch’s *t*-test was used for two-group comparisons because it does not assume equal variances across groups and is therefore more appropriate under unequal sample-size conditions. For multi-group performer-level comparisons, one-way ANOVA was reported together with the non-parametric Kruskal–Wallis test as a robustness check, given the small and unequal group sizes. Effect sizes were reported using Cohen’s d where applicable.

The reported *p*-values should be understood as nominal descriptive indicators of within-corpus score separation, not as evidence for population-level effects or as validation of stylistic similarity. No alpha threshold was used to make binary significance decisions, and no single *p*-value is treated as sufficient evidence of stylistic change, historical influence, or training effect. Because the study involves several related comparisons across historical stages, reference genres, and performer groups, no formal correction for multiple comparisons was applied. This decision reflects the exploratory and descriptive role of the statistical summaries, rather than an attempt to support confirmatory inference.

Accordingly, interpretation is based primarily on the convergence of several forms of evidence: the direction of model-derived score differences, effect-size estimates, distributional patterns, corpus composition, performer background, and the case-based acoustic comparison. Statistical summaries are used only to describe whether patterned differences appear within the present corpus. They do not by themselves establish causal effects of training background, stable population-level differences among historical stages, or validated stylistic similarity between genres.

For the performer-level analysis of Yueju- and Wuju-related model-derived scores (Section 3.1.3), both parametric and non-parametric results are treated as exploratory and descriptive. These tests are used to identify patterned variation within the present corpus, not to establish causal effects of training background or population-level generalizations. This caution is especially important because several performer-level groups contain small numbers of excerpts.

Given the small size of the Traditional Sanjiaoxi corpus (*n* = 20), comparisons involving this corpus should be interpreted with particular caution. Statistical outcomes in these comparisons should not be read as establishing stable population-level differences among historical stages. Rather, the tests are used descriptively to assess whether the observed model-derived score differences are patterned within the present corpus. The implications of this sample-size limitation are discussed in Section 4.4.

## 3 Results

### 3.1 Exploratory model-derived acoustic-proximity results: diachronic change in Muju style

The following three subsections (3.1.1–3.1.3) report results from the exploratory score-based acoustic-proximity analysis. As noted in Section 2.2.1, the reported values should be interpreted as model-derived scores of relative acoustic proximity within the learned acoustic feature space, rather than as classification labels or validated measures of stylistic similarity. This acoustic-level interpretation is used only as a basis for discussing vocal-production tendencies, because vocal timbre, articulation, resonance, and habitual singing features are understood by Muju practitioners and local insiders as audible components of genre style.

#### 3.1.1 Comparison with the Traditional Sanjiaoxi corpus

When the Old Muju corpus was compared with the Traditional Sanjiaoxi corpus, the mean model-derived proximity score was 83.76% (*SD* = 11.14). The Contemporary Muju corpus showed a lower mean score of 76.33% (*SD* = 11.31). The comparison showed a clear within-corpus score separation between the two later corpora in their model-derived proximity to Traditional Sanjiaoxi, with a low nominal *p*-value (*p* < 0.001, Welch’s *t* = 4.38) and a medium descriptive effect size (Cohen’s *d* = −0.66).

The distributional pattern is consistent with the same tendency. In the Old Muju corpus, 96.7% of excerpts exceeded 60, 94.5% exceeded 70, 64.8% exceeded 80, and 30.8% exceeded 90% on this model-derived index. In the Contemporary Muju corpus, the corresponding proportions were 89.4, 72.9, 43.5, and 12.9%, respectively. Within the present modeling framework, these results are consistent with the possibility that Contemporary Muju is less closely aligned with Traditional Sanjiaoxi than Old Muju is (Table 3).

**Table 3:** Model-derived acoustic-proximity scores of later Muju corpora to the Traditional Sanjiaoxi corpus.

Corpus	n	Mean acoustic-proximity score (%)	SD	Proportion >60%	Proportion >70%	Proportion >80%	Proportion >90%
Old Muju	91	83.76	11.14	96.7	94.5	64.8	30.8
Contemporary Muju	85	76.33	11.31	89.4	72.9	43.5	12.9

Values are model-derived acoustic-proximity scores within the learned feature space. They are interpreted as exploratory indicators of relative acoustic proximity, not as direct measures of genre identity or validated stylistic similarity. The group comparison between Old Muju and Contemporary Muju was conducted using Welch’s t-test. Old Muju showed significantly higher scores than Contemporary Muju (*p* < 0.001, Welch’s *t* = 4.38, Cohen’s *d* = −0.66).

#### 3.1.2 Cross-genre proximity across historical stages

In the three-way comparison with Huangmeixi, Yueju, and Wuju, the Traditional Sanjiaoxi corpus (*n* = 20) yields mean model-derived scores of 61.05% for Huangmeixi, 31.27% for Yueju, and 7.68% for Wuju. In the Old Muju corpus (*n* = 91), the mean Huangmeixi-related score increased to 85.53%, while the Yueju- and Wuju-related scores decreased to 13.57 and 0.91%, respectively. In the Contemporary Muju corpus (*n* = 85), the corresponding means are 89.86% for Huangmeixi, 9.49% for Yueju, and 0.65% for Wuju.

Relative to the Traditional Sanjiaoxi corpus, the Huangmeixi-related model-derived score was higher in both the Old Muju corpus and the Contemporary Muju corpus, with low nominal *p*-values in the exploratory statistical summaries (both *p* < 0.001). A similar tendency appeared in the highest-scoring category among the three reference genres. Huangmeixi received the highest model-derived score for 75.0% of excerpts in the Traditional Sanjiaoxi corpus. The corresponding proportions were 95.6% in the Old Muju corpus and 98.8% in the Contemporary Muju corpus. These results suggest that, within the present modeling framework, Huangmeixi-related model-derived scores become more concentrated across the later historical stages of Muju. They should not, however, be taken as direct proof that Muju “became Huangmeixi” (Table 4).

**Table 4:** Huangmeixi-related model-derived scores across the three historical stages of Muju.

Corpus	n	Mean Huangmeixi-related score (%)	SD	Median	Min	Max	Huangmeixi as highest-scoring category (%)	Huangmeixi-related score >70%	Huangmeixi-related score >90%
Traditional Sanjiaoxi	20	61.05	18.78	63.31	24.78	89.32	75.0%	35.0%	0.0%
Old Muju	91	85.53	13.86	89.72	42.44	99.95	95.6%	82.4%	61.5%
Contemporary Muju	85	89.86	11.17	94.46	48.56	99.97	98.8%	94.1%	68.2%

Values indicate Huangmeixi-related model-derived acoustic-proximity scores for each historical stage of Muju. Higher values indicate stronger association with the learned Huangmeixi-related acoustic pattern within the model’s feature space.

#### 3.1.3 Performer training background and within-corpus variation

To examine whether performer training background may be associated with variation within the Contemporary Muju corpus, the data were disaggregated by performer. Among the five focal performers, the two performers most closely associated with Yueju exposure (P3 and P4) show the highest Yueju-related model-derived scores. P3 yields a mean Yueju-related score of 11.65%, and P4 yields 20.11%. Both values were above the five-performer mean of 9.16%. The exploratory performer-level comparison indicated patterned variation in Yueju-related scores across the five focal performers (one-way ANOVA: *F* = 4.96, *p* = 0.002; Kruskal–Wallis: *p* = 0.001) (Table 5).

**Table 5:** Yueju-related model-derived scores for the five focal performers in the contemporary Muju corpus.

Performer or group	n	Mean Yueju-related score (%)
Contemporary Muju overall	85	9.49
P3	12	11.65
P4	6	20.11
Mean across five focal performers	51	9.16
Non-Yueju-trained focal performers	33	6.26

Values indicate Yueju-related model-derived acoustic-proximity scores for the five focal performers in the Contemporary Muju corpus. Performer identity was not used as a model input or classification label. The scores are used descriptively to examine within-corpus variation and should not be interpreted as definitive evidence of training effects.

It should be noted that P4, although recorded as having received no formal professional training in Yueju, exhibited the highest mean Yueju-related model-derived score among the five focal performers (20.11%). Several factors may contribute to this pattern. P4 is from Ningbo, Zhejiang, a region where Yueju has a deep local presence. Moreover, formal institutional training does not exhaust all forms of genre exposure in Chinese opera practice, where self-directed learning and long-term local immersion may also shape vocal habits. Additionally, P4 contributed only six excerpts to the corpus, and estimates based on small samples are inherently less stable. This observation underscores the exploratory nature of the performer-level analysis and cautions against treating these scores as definitive evidence of training effects.

A similar but weaker pattern was observed for Wuju. The Wuju-trained performer P5 yielded a mean Wuju-related score of 0.69%, which was slightly higher than the overall corpus mean of 0.65% and higher than the five-performer mean of 0.32%. The comparison between P5 and the remaining performers suggested a possible tendency but did not provide stable confirmatory evidence (Welch’s *t* = 2.24, *p* = 0.051, Cohen’s *d* = 0.89) (Table 6).

**Table 6:** Wuju-related model-derived scores for the five focal performers in the contemporary Muju corpus.

Performer or group	n	Mean Wuju-related score (%)
Contemporary Muju overall	85	0.65
P5	8	0.69
Mean across five focal performers	51	0.32
Non-Wuju-trained focal performers	43	0.28

Values indicate Wuju-related model-derived acoustic-proximity scores for the five focal performers in the Contemporary Muju corpus. Performer identity was retained only as metadata for interpretation. The scores are exploratory and should not be interpreted as establishing a causal relation between training background and vocal style.

These performer-level patterns should be interpreted cautiously. They suggest that model-derived scores vary in ways broadly consistent with performer training background, but they do not by themselves establish a direct causal relation between training history and vocal style.

#### 3.1.4 Diagnostic model metrics under the present corpus partitioning

To provide a basic diagnostic check of the learned acoustic feature space, model metrics were computed through binary genre-discrimination tasks performed separately on the three reference genres with the largest sample sizes (Huangmeixi, Wuju, and Yueju). In each task, the model was trained to distinguish one target reference genre from the combined set of all other excerpts. Table 7 report training loss, validation accuracy, and test accuracy monitored up to epoch 20 for each binary task. These diagnostic runs were extended beyond the epoch-15 checkpoint used in the main score-based analyses in order to examine whether additional fine-tuning improved performance.

**Table 7:** Model evaluation metrics for binary genre-discrimination tasks.

Epoch	Huangmeixi	Wuju	Yueju
A. Training loss (binary classification per genre).			
1	0.719	1.282	0.968
5	0.625	0.886	0.732
10	0.573	0.659	0.570
15	0.527	0.479	0.498
20	0.536	0.492	0.514
B. Validation accuracy (binary classification per genre).			
1	62.6%	32.2%	44.3%
5	67.8%	49.3%	59.5%
10	70.5%	61.9%	69.8%
15	75.1%	74.9%	74.9%
20	74.8%	72.4%	73.3%
C. Test accuracy (binary classification per genre).			
1	64.9%	30.9%	41.2%
5	73.2%	49.5%	66.0%
10	73.2%	61.9%	73.2%
15	75.3%	75.1%	80.4%
20	74.4%	71.7%	77.5%

Evaluation was monitored up to epoch 20 to assess convergence and checkpoint selection. The main score-based analyses used the epoch-15 checkpoint, where validation and test accuracy had already stabilized and no further improvement was observed at epoch 20.

All three binary tasks showed decreasing training loss and improved validation/test accuracy during fine-tuning. By epoch 15, test accuracy ranged from 75.1 to 80.4%. The additional epoch-20 results did not improve on the epoch-15 test accuracy, supporting the use of the epoch-15 checkpoint for the main score-based analyses. These metrics indicate that, under the present corpus partitioning, the model was able to learn acoustic patterns that contributed to genre discrimination among the reference corpora.

However, these metrics should be interpreted cautiously. As described in Section 2.2.3, the data split was stratified at the genre-label level rather than at the performer level. Therefore, the reported validation and test metrics should not be interpreted as evidence of speaker-independent classification performance. They also do not resolve the possibility that performer-specific vocal characteristics contributed to the learned feature space.

For this reason, the evaluation metrics and the acoustic-proximity scores serve different purposes. The diagnostic metrics provide a basic check that the model can learn genre-discriminative acoustic patterns under the present corpus conditions. The model-derived acoustic-proximity scores reported in Sections 3.1.1–3.1.3 are then used comparatively to examine how strongly target excerpts are associated with learned genre-related acoustic patterns within the same feature space. These scores are not treated as direct measures of stylistic similarity, and the diagnostic accuracy values are not presented as a comprehensive benchmark for an optimized genre-classification system.

### 3.2 Acoustic case analysis

The following acoustic case analysis presents four representative matched syllables—“*bai*,” “*nian*,” “*shuang*,” and “*xin*.” These syllables were selected because they show clear correspondence across the three performances and cover different syllabic and articulatory contexts. The analysis proceeds in two steps. The first comparison, between P1 and P6, retains the historical reference dimension of the case study. The second comparison, between P1 and P7, provides the central contemporary contrast for examining whether P1’s vocal production shows patterns compatible with possible effects of prior Huangmeixi training.

#### 3.2.1 Comparison between P1 and P6

The P1–P6 comparison provides a historical reference for situating P1’s contemporary vocal production. P6 is not treated as a directly matched control because the two recordings differ in gender, historical period, and recording condition. The cleaned raw formant values show that P1 and P6 differ across multiple syllables, but not in a uniform F1 direction. P1 showed lower F1 than P6 in “*bai*” and “*shuang*,” nearly identical F1 in “*nian*,” and higher F1 in “*xin*.” This indicates that the P1–P6 contrast cannot be reduced to a single difference in vowel openness.

**Figure 2:**
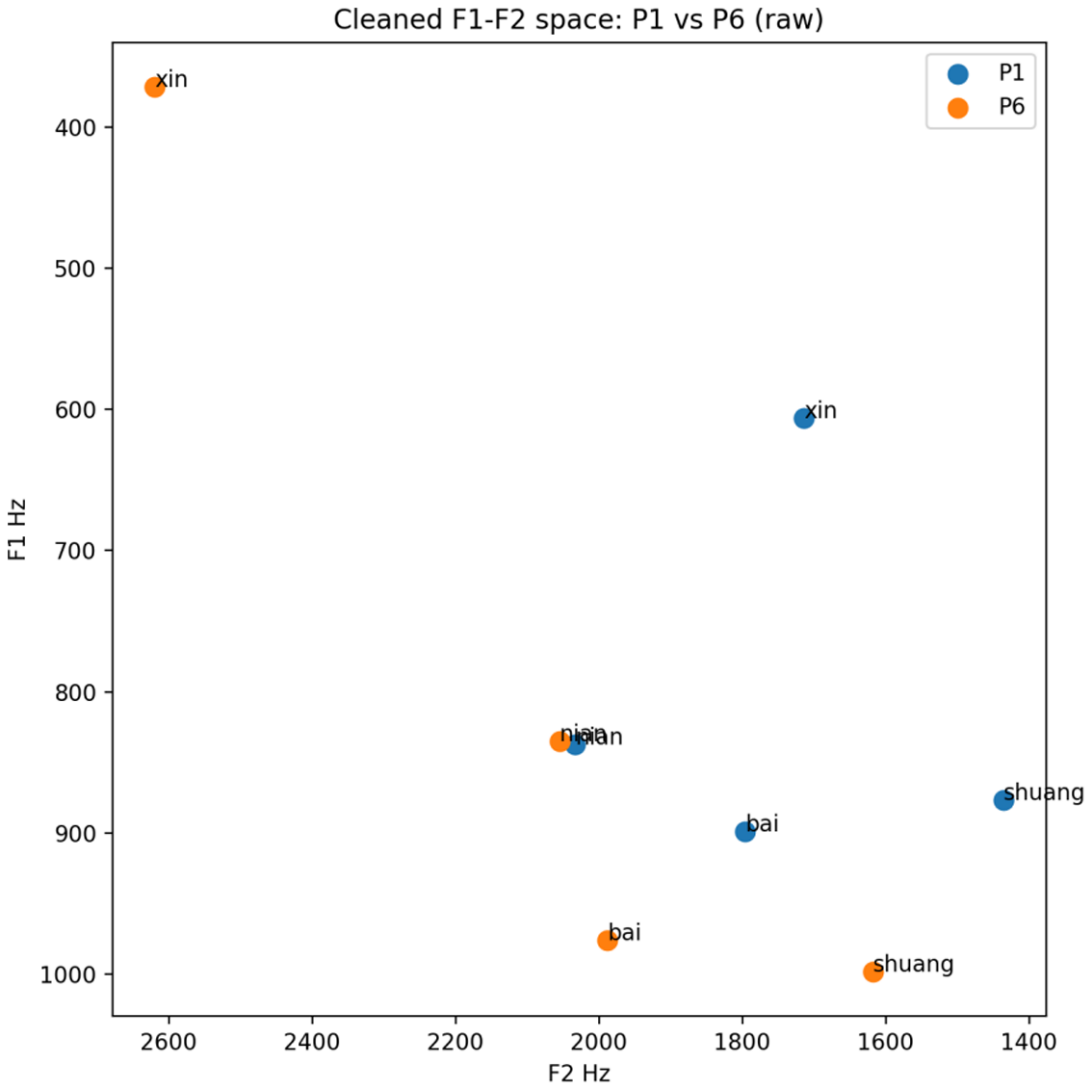
Cleaned raw F1–F2 comparison between P1 and P6. The figure shows the positions of “*bai*,” “*nian*,” “*shuang*,” and “*xin*” in the cleaned raw F1–F2 space for P1 and P6. P6 is used as a historical Muju/Sanjiaoxi reference, whereas P1 represents a contemporary Huangmeixi-trained Muju performer. The values were recalculated from frame-level formant data after retaining the central 60% of each syllable segment and excluding implausible formant values. Both axes are plotted in the conventional vowel-space orientation, with lower F1 toward the top and higher F2 toward the left. This comparison is intended as a historical reference rather than as a gender-matched control comparison.

The F2 results show a clearer contrast. P1 showed lower raw F2 than P6 in all four syllables, with the strongest difference in “*xin*” (P1 = 1715 Hz; P6 = 2,631 Hz). Because F2 is broadly associated with the front-back dimension of vowel articulation, this pattern suggests a relatively more backed articulatory placement in P1 than in the historical reference. After within-singer normalization, this contrast remained most visible in “*xin*,” where P1 showed much lower normalized F2 than P6 (P1 = −0.229; P6 = 1.559). Because the P1–P6 comparison remains affected by gender and historical recording conditions, it is treated here as historically informative rather than as direct evidence of training transfer. Therefore, the P1–P6 comparison is used only to situate P1 against the earliest available historical reference, not to isolate training effects (Figure 2).

#### 3.2.2 Comparison between P1 and P7

The P1–P7 comparison is more central to the argument of this section. P7 is a Chun’an local female performer who did not receive professional opera-school training and is used here as a contemporary same-gender local reference for Muju vocal practice. Compared with the P1–P6 contrast, the P1–P7 comparison is better suited to examining possible training-related vocal-production differences, because both singers are contemporary female performers while differing in training background and local affiliation.

The cleaned raw F1 values show a consistent contrast between P1 and P7. P1 showed higher F1 than P7 in all four selected syllables (Table 8). Since F1 is broadly associated with vowel openness, this repeated pattern suggests that P1 tended toward a more open vowel realization than the Chun’an local reference. The consistency of this pattern is important: after unstable boundary frames and implausible formant values were removed, P1 still showed higher F1 across all four syllables.

**Table 8:** Cleaned raw F1 and F2 values for P1 and P7 across selected syllables.

Syllable	P1 F1 (Hz)	P7 F1 (Hz)	P1 F2 (Hz)	P7 F2 (Hz)	Main observation
*bai*	892	764	1782	1926	P1 higher F1, lower F2
*nian*	837	677	2033	2,268	P1 higher F1, lower F2
*shuang*	877	762	1,438	1,171	P1 higher F1, higher F2
*xin*	596	317	1715	2,489	P1 higher F1, lower F2

Values are rounded means calculated from cleaned frame-level formant data after retaining the operationally defined central portion of each syllable segment and excluding implausible formant values. F1 is used as an acoustic indicator of vowel openness, and F2 is used as an indicator of relative articulatory fronting/backing. The comparison is descriptive and case-based; it is not used for population-level inference.

**Figure 3:**
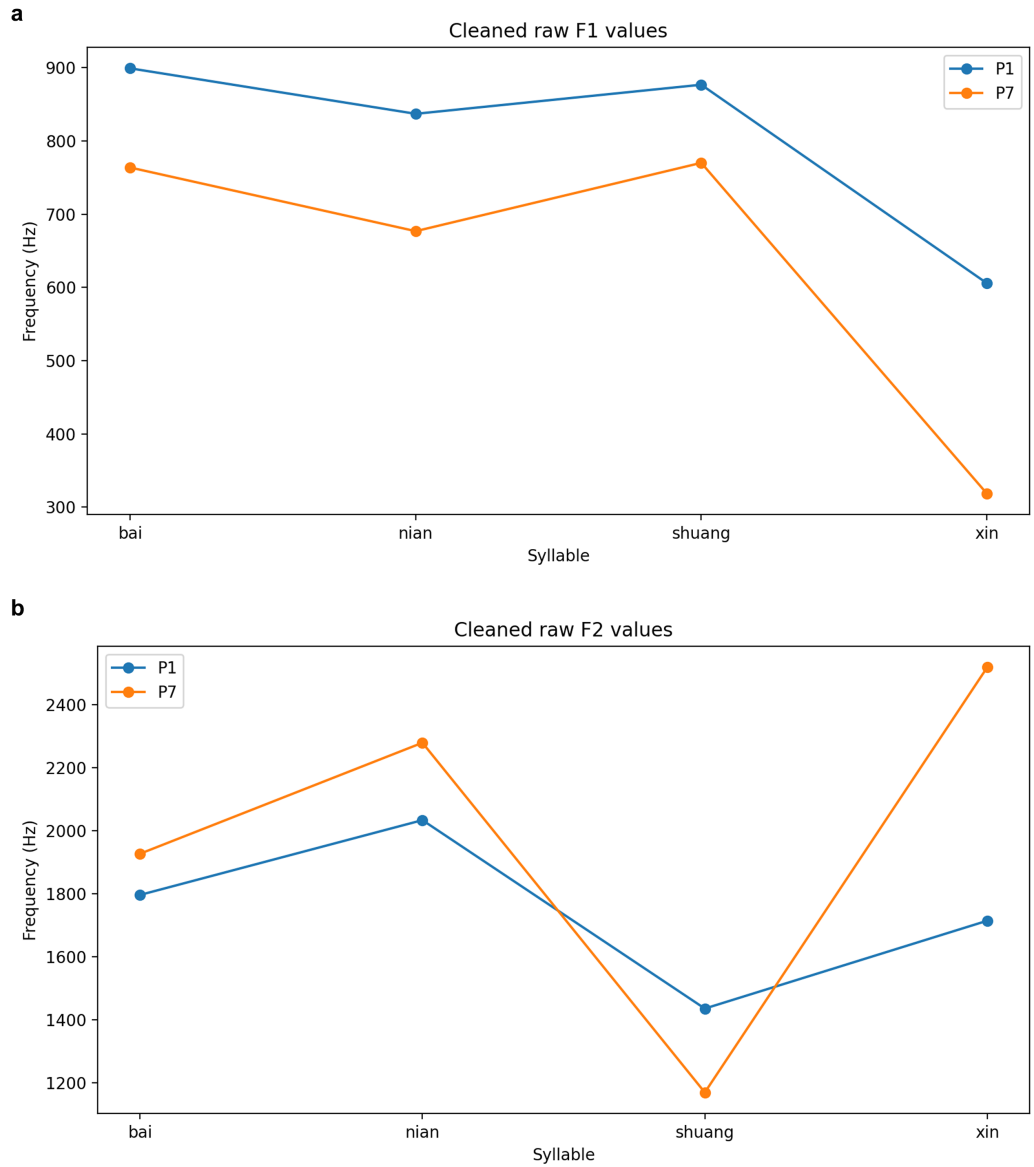
Cleaned raw F1 and F2 comparison between P1 and P7. **(a)** Cleaned raw F1 values. **(b)** Cleaned raw F2 values. The figure visualizes the cleaned raw F1 and F2 patterns reported in Table 8. Values are means calculated from cleaned frame-level formant data after retaining the central 60% of each syllable segment and excluding implausible formant values.

The F2 values further suggest differences in articulatory placement. P1 showed lower F2 than P7 in three of the four syllables (Table 8). This suggests that P1 tended toward a relatively backed articulatory placement in several syllabic contexts. The only exception was “*shuang*,” where P1 showed higher F2 than P7 (P1 = 1,436 Hz; P7 = 1,171 Hz). This exception indicates that the difference between P1 and P7 should not be described as a uniform shift across all syllables, but rather as a recurrent tendency shaped by syllabic and phonetic context (Figure 3).

**Figure 4:**
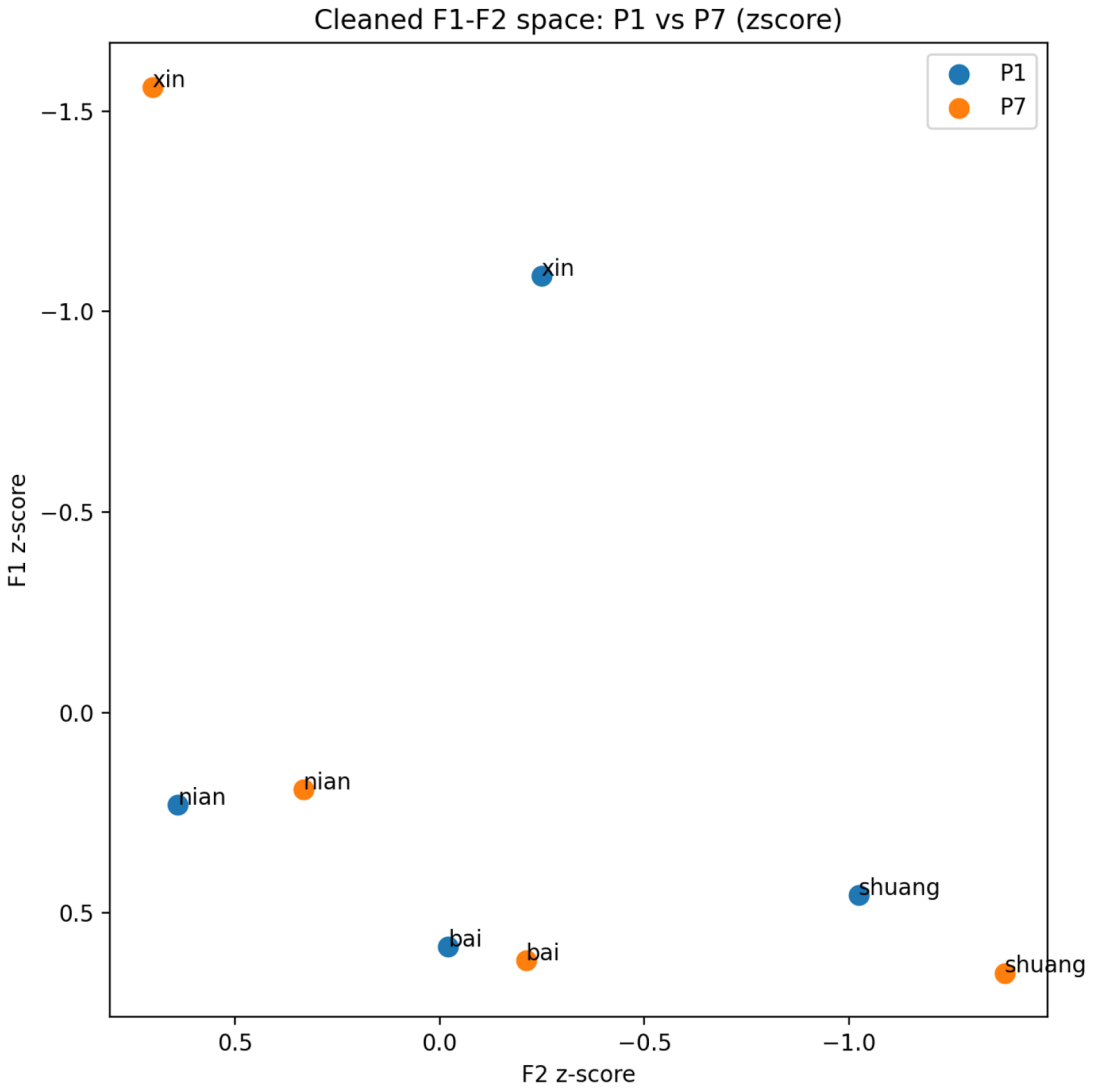
Within-singer normalized F1–F2 comparison between P1 and P7. The figure shows the four selected syllables in the within-singer normalized F1–F2 space. Normalized values were calculated as z-scores separately for each singer and each formant dimension. The clearest P1–P7 contrast appears in “*xin*,” where P1 shows relatively higher F1 and lower F2 than P7 after normalization. The normalized values are used as a descriptive sensitivity check. Both axes are plotted in the conventional vowel-space orientation, with lower F1 toward the top and higher F2 toward the left.

The normalized results provide a descriptive sensitivity check rather than an independent test of stylistic difference. The clearest contrast appears in “*xin”*: P1 had higher normalized F1 than P7 (P1 = −1.102; P7 = −1.551) and lower normalized F2 than P7 (P1 = −0.229; P7 = 0.677). After reducing singer-specific scaling effects, P1 therefore still showed a more open and relatively more backed realization of this syllable. This pattern is compatible with the possibility that prior Huangmeixi training contributed to P1’s vocal-production habits, although it cannot by itself establish a causal training effect (Figure 4).

#### 3.2.3 Summary of the case comparison

The two comparisons together clarify the performance-level implications of the acoustic case analysis. The P1–P6 comparison preserves the historical reference dimension, but its interpretation remains limited by differences in gender, recording period, and recording condition. It is therefore used to situate P1 against the earliest available Muju/Sanjiaoxi reference, rather than to isolate the effect of training background.

The P1–P7 comparison provides a more focused same-gender contemporary contrast. In the cleaned raw values, P1 shows consistently higher F1 than the Chun’an local female reference across the four selected syllables, and lower F2 in three of the four syllables. This pattern suggests a recurrent tendency toward more open vowel realization and relatively backed articulatory placement, while the exception in “*shuang*” indicates that the contrast is also shaped by syllabic and phonetic context. The within-singer normalized results further support this interpretation most clearly in “*xin*,” but they should be treated as a descriptive sensitivity check rather than as independent statistical evidence.

These findings are consistent with the idea of reconfiguration rather than replacement. P1’s singing remains within Muju performance, but the observed F1 and F2 patterns are compatible with the possibility that prior Huangmeixi training contributed to her vocal-production habits. Cross-genre training may therefore enter contemporary Muju not only as an external biographical fact, but also through embodied vocal habits that shape vowel realization and articulatory placement. Because the comparison is based on a small number of singers and selected syllable excerpts, these findings should be treated as suggestive rather than conclusive.

## 4 Discussion

### 4.1 Nature of change

The central argument of this study is not that contemporary Muju has been replaced by another genre, but that its vocal profile may have been reconfigured under contemporary performance conditions. Within the present analytical framework, both Old Muju and Contemporary Muju remain connected to Traditional Sanjiaoxi, while Huangmeixi-related model-derived scores become more concentrated in the later stages.

What appears to have changed, therefore, is not historical continuity as such, but the internal balance of stylistic relations within Muju. The present results suggest that a historically available axis of affinity has been selectively intensified under contemporary conditions. Because the AI-assisted findings remain exploratory, however, this point should be treated as a hypothesis supported by converging indications rather than as a definitive demonstration.

From a performance-science perspective, such a pattern is compatible with *positive transfer* in motor learning, whereby previously acquired skills can facilitate performance in a related task without simply reproducing the source practice in unchanged form (Schmidt et al., 2019). In the Muju case, Huangmeixi-trained performers did not necessarily reproduce Huangmeixi singing directly; rather, the results are consistent with the possibility that they performed Muju repertoire through vocal habits shaped by prior Huangmeixi training.

The acoustic case comparison discussed in Section 3.2 does not establish this mechanism conclusively, but it does provide a focused illustration of how training-related differences may become audible at the level of vocal production.

### 4.2 Cross-genre training as a mechanism of vocal change

Interpreted through the framework of training transfer in embodied vocal production, this reconfiguration may operate through two related factors: performer mobility and habitual vocal practice. The performer-level findings do not prove that training background determines contemporary Muju style. However, they suggest that model-derived scores vary in ways broadly compatible with differences in prior training. The acoustic case analysis further illustrates how training transfer may operate at the level of embodied vocal production. The comparison between P1 and P7 shows that the Huangmeixi-trained performer differs from the Chun’an local reference mainly in vowel openness and articulatory placement. This suggests that cross-genre training may enter Muju not simply as an external biographical fact, but through habitual ways of shaping vowels and articulating syllables.

This interpretation is consistent with established principles in the study of vocal expertise. Research on singing has shown that trained singers develop habitual patterns of vocal-tract configuration, laryngeal control, and resonance adjustment that become automatic through extensive practice (Sundberg, 1987; Titze, 2000; Omori et al., 1996). In the language of motor learning theory, the vocal habits formed through years of genre-specific training function as *overlearned motor schemas*—stable, highly practiced movement patterns that persist even when the performance context changes (Schmidt et al., 2019; Magill and Anderson, 2016). The present findings do not demonstrate this process directly, but they are compatible with such an account.

Field materials further contextualize this mechanism. As one of the leaders of the troupe’s reorganization recalled, “Most local young people in Chun’an were unwilling to come. Parents preferred their children to focus on academics rather than doing this [acting]” (field interview, November 26, 2021, Huoluzhai Peak, Chun’an County). This statement suggests that the contemporary troupe was rebuilt under conditions in which locally transmitted personnel alone could no longer sustain stage production. In this sense, the incorporation of cross-genre-trained performers may have been not merely a consequence of stylistic change, but part of the institutional setting within which such change became possible.

### 4.3 Vocal production as a measurable dimension of stylistic change

The Muju case suggests that change in a performing-art tradition can be examined not only at the level of repertoire or institutional history, but also at the level of vocal production. This point is important because changes in performing traditions are often described through troupe history, repertoire reform, or performer recruitment, whereas the voice itself—that is, how sound is physically produced—has often received less direct analytical attention.

In operatic singing, style is realized through concrete vocal practice: how singers configure the vocal tract, place articulation, organize resonance, and shape timbre. Recent work on Peking opera has shown that vocal timbre can serve as an analytically meaningful dimension of role and style differentiation (Liu and Wallmark, 2024). Recent work on Cantonese operatic singing has also proposed and piloted explicit assessment frameworks for pedagogy, suggesting that the formalization of vocal training is an important dimension in at least some contemporary Chinese opera teaching contexts (Luo and Leung, 2023).

The present study does not offer a definitive measurement of stylistic change. Its contribution is to propose an exploratory framework for tracing possible training-related differences in vocal production. This framework operates at two levels: model-derived corpus-level patterns and focused case-level acoustic comparison. The value of this two-level approach lies in bringing corpus-scale patterning and case-level acoustic observation into the same analytical conversation, while keeping their evidential weight distinct. Theoretically, this approach reframes local vocal style as an embodied and measurable dimension of performance practice, rather than only as a repertoire category, regional label, or insider description.

The finding that vocal style may be reconfigured through training transfer also has practical implications. For heritage practitioners, it suggests that sustaining a genre’s distinctive vocal quality may require attention not only to repertoire transmission but also to the training pathways through which performers enter the tradition. In the Muju case, the model-derived scores suggest that Huangmeixi-related tendencies are especially prominent among the cross-genre influences examined, although this observation remains exploratory and should not be overgeneralized.

### 4.4 Limitations and future directions

Several limitations should be noted.

First, the Traditional Sanjiaoxi corpus is limited to 20 excerpts, creating a substantial imbalance relative to the Old Muju and Contemporary Muju corpora. As explained in Section 2.1, this limitation reflects the scarcity of surviving audio materials for a rare and under-documented local opera tradition rather than a selective sampling decision. Nevertheless, because Traditional Sanjiaoxi functions as a key historical reference point in several comparisons, the limited corpus size may affect the stability of model-derived scores. Under such conditions, results involving Traditional Sanjiaoxi may be more sensitive to individual recordings, and observed cross-period differences should therefore be interpreted as preliminary corpus-level tendencies rather than stable estimates of diachronic change. Future research should expand the historical corpus where possible and may also complement audio-based comparison with archival documents, notated materials, and further field-based evidence.

Second, the AI-assisted score-based analysis remains exploratory in an important methodological sense. The model-evaluation results in Section 3.1.4 show that the model learned genre-discriminative acoustic features under the present corpus conditions. Test accuracy ranged from 75.1 to 80.4% across the three binary evaluation tasks involving Huangmeixi, Wuju, and Yueju. These results provide a basic diagnostic check for the learned acoustic feature space under the present corpus partitioning.

However, the model was not designed as a standalone genre-classification system, and classification accuracy is not the final object of analysis in this study. The model-derived scores are therefore treated as exploratory indicators of relative acoustic proximity within the learned feature space, rather than as direct or validated measures of stylistic similarity. They can indicate patterned acoustic tendencies across corpora, but they do not by themselves establish historical influence, genre identity, or causal mechanisms of stylistic change. The associated statistical tests should therefore be understood as descriptive tools for identifying patterned differences in model-derived scores, not as confirmatory tests establishing population-level stylistic change.

Third, a related limitation concerns data partitioning and possible performer-level leakage. In both pre-training and fine-tuning, stratification was conducted at the genre-label level rather than at the performer level, meaning that individual performers could in principle appear across train, validation, and test splits. Performer identity was not used as a model input or classification label, but performer-specific vocal characteristics cannot be completely ruled out as part of the learned acoustic space. This limitation affects the evidential weight of the diagnostic accuracy metrics and the performer-level analysis. The reported validation and test metrics should therefore not be interpreted as evidence of speaker-independent classification performance. For the reference corpora, which draw from published recordings by many performers across a broad historical span, the risk of systematic performer-level leakage dominating genre-level signal is limited. For the smaller Muju corpus, however, performer identity and genre membership are more closely intertwined. Future work should consider performer-level cross-validation when larger and more balanced corpora become available.

Fourth, the interpretability of the model remains limited. Transformer-based scores do not directly reveal which acoustic features drive particular score tendencies. As a result, the present analysis can identify patterned relations in model-derived scores, but it cannot by itself explain which specific acoustic properties are responsible for those relations. Future work incorporating interpretability methods may help clarify this issue.

Fifth, the acoustic case analysis remains limited by the structure of the available materials. Although the addition of P7 provides a contemporary same-gender local reference for comparison with P1, the historical reference comparison between P1 and P6 still involves differences in gender, recording period, and recording condition. The P1–P7 comparison reduces but does not eliminate interpretive risk, because it is still based on a small number of singers and selected syllable excerpts. The case findings should therefore be treated as suggestive rather than conclusive.

Sixth, the findings are specific to Muju and cannot be generalized directly to other opera genres without further investigation. However, the analytical framework—combining exploratory corpus-level modeling with focused case-level acoustic comparison—may be useful for studying other performing traditions undergoing similar processes of reconstitution through cross-trained performers.

## 5 Conclusion

This study examined how the local vocal characteristics of Muju may be reconfigured in contemporary performance following the incorporation of performers trained in other Chinese opera genres. Using exploratory score-based AI-assisted analysis and a case-based acoustic analysis, it reported three broad tendencies.

First, the model-derived corpus-level scores are consistent with the possibility that Contemporary Muju shows weaker relative alignment with Traditional Sanjiaoxi than Old Muju does. Second, Huangmeixi-related model-derived scores appear to become more concentrated across the later historical stages of Muju. This pattern is compatible with, but does not by itself prove, the influence of cross-genre training.

Third, the acoustic case comparison suggests that performers with different training backgrounds may exhibit recurrent differences in vocal production, especially in vowel openness and articulatory placement. In the revised case design, P6 provides a historical Muju/Sanjiaoxi reference. The P1–P7 comparison offers a more focused contemporary same-gender contrast between a Huangmeixi-trained performer and a Chun’an local female reference.

These three observations carry different evidential weight. The acoustic case study offers a focused illustration of how possible training-related differences may become audible in performance, but it does not establish such differences conclusively. The P1–P7 comparison reduces the interpretive risk associated with the original male–female historical comparison. However, it remains based on a small number of singers and selected syllable excerpts. The AI-assisted score-based analysis provides an exploratory corpus-level perspective on broader diachronic tendencies. Its interpretive value remains limited by the indirect relation between model-derived acoustic-proximity scores and stylistic similarity. The metrics reported in Section 3.1.4 confirm that the model reliably learns genre-discriminative acoustic features, but they do not turn these scores into direct measures of stylistic similarity.

Taken together, the findings are consistent with the view that stylistic change in a traditional performing art can be investigated empirically at the level of vocal production, while still requiring careful attention to evidential limits. In the Muju case, the results support the possibility that when cross-trained performers enter a new genre tradition, previously acquired vocal habits may shape the resulting vocal profile in observable ways.

For performance-science research, this study offers a naturalistic example of how training transfer may be explored in vocal production. For heritage practice, it suggests that the sonic identity of a genre may depend not only on repertoire and institutions, but also on the training pathways through which performers come to inhabit the tradition.

## Data Availability

The dataset analyzed in this study was not made fully public because it contained undisclosed audio recordings of archives as well as live recordings of identifiable performers, which were subject to copyright restrictions, archive access regulations, and ethical requirements. Requests to access the datasets should be directed to Yijia Cao, cyjsophie@126.com.
